# Features-Based Deisotoping Method for Tandem Mass Spectra

**DOI:** 10.1155/2011/210805

**Published:** 2012-01-04

**Authors:** Zheng Yuan, Jinhong Shi, Wenjun Lin, Bolin Chen, Fang-Xiang Wu

**Affiliations:** ^1^Division of Biomedical Engineering, University of Saskatchewan, Saskatoon, SK, Canada S7N5A9; ^2^Department of Mechanical Engineering, University of Saskatchewan, Saskatoon, SK, Canada S7N5A9

## Abstract

For high-resolution tandem mass spectra, the determination of monoisotopic masses of fragment ions plays a key role in the subsequent peptide and protein identification. In this paper, we present a new algorithm for deisotoping the bottom-up spectra. Isotopic-cluster graphs are constructed to describe the relationship between all possible isotopic clusters. Based on the relationship in isotopic-cluster graphs, each possible isotopic cluster is assessed with a score function, which is built by combining nonintensity and intensity features of fragment ions. The non-intensity features are used to prevent fragment ions with low intensity from being removed. Dynamic programming is adopted to find the highest score path with the most reliable isotopic clusters. The experimental results have shown that the average Mascot scores and *F*-scores of identified peptides from spectra processed by our deisotoping method are greater than those by YADA and MS-Deconv software.

## 1. Introduction

With the development of tandem mass spectrometry, it has obtained an important status in protein and peptide analysis, such as the acquisition of structure information and identification and qualitative analysis [1]. Since the fundamental data used for peptide identification in tandem mass spectra (MS/MS) is the *m*/*z* values, charge states of fragment ions, their detection can directly influence the subsequent analysis of mass spectra including the peptide identification and quantification [2]. However, there are two difficulties during the process of detecting fragment ions: first, in some cases many real fragment ions have very low intensity that they can be removed as noise peaks by accident [3]. Numerous noisy peaks in tandem mass spectra can cause either false negative or false positive fragment ions. Second, due to the existence of heavy isotopes in nature, more than one isotopic peak for each fragment ion is resolved in high-resolution tandem mass spectra. Though isotopic peaks can provide us useful information, such as compound composition and charge states, it will cost an expensive computation if peptide identification is done without removing them. And, also, isotopic peaks can overlap that could result in wrong interpretation of masses of fragment ions. Thus, to increase the accuracy of the peptide identification and reduce the complexity of MS/MS analysis, many existing deisotoping algorithms [4–19] have already been explored to detect the isotopic clusters of fragment ions.

Some of these deisotoping methods [4–10, 19] are based on the theoretical isotopic distribution matching with experimental isotopic distribution. And the theoretical isotopic distribution can be estimated according to the monoisotopic mass of peptide ions [5, 17, 20, 21]. If the observed signals matched well with the theoretical isotopic distribution, then these signals will be considered as isotopic clusters and be subtracted from the spectrum. This procedure will be repeated until no more possible isotopic clusters can be found. THRASH [5], one of the most well-known algorithms, is adapted by several algorithms, such as Decon2LS [8] and ICR2LS [10]. This algorithm is performed as follows: determination of noise intensity level; charge state determination by Fourier-Transform/Patterson techniques; estimation of the composition of the peptide ions based on the average amino acid Averigine [11]; calculation of theoretical isotopic distribution; matching theoretical isotopic distribution with the experimental one by the least-squares fitting to identify the monoisotopic peaks. However, the overlapping signal peaks in MS/MS always happen, leading to an expensive cost. Thus, the major shortage of this template matching is that, in case of overlapping clusters, it is not effective enough to identify the isotopic clusters only based on the intensity information of theoretical isotopic distribution and experimental isotopic distribution. Once one isotopic envelop is incorrectly identified, the determination of the isotopic envelop behind will easily get wrong, like error propagation. 

Li et al. [12] proposed a quadratic programming deisotoping approach called Pepex in which observed spectra are modeled by a linear mixture model. Given theoretical isotopic distribution and the observed isotopic distribution, the lowest number of peptides which can well explain the observed spectrum needs to be determined by solving a quadratic programming problem. But, in this method, many parameters need to be optimized and it is limited to the dataset with single charge. 

Samuelsson et al. [13] formulate the deisotoping issue into the statistical problem of variable selection. This method selects the simplest model with the least number of isotopic clusters that can interpret MS/MS well. Du's method avoids greedy feature selection as well. However, it is not justified to select the least number of isotopic clusters from the spectrum. Though this criterion can decrease the false positives and false negatives, the sensitivity is also reduced. 

With the exception of these algorithms above, Du and Angeletti [14] developed a nonlinear parametric model for the *m*/*z* interval of 1 Dalton. And, then, they used Bayesian method to estimate the probabilities of the signal peak of an ion and the parameters of the model. For each signal peak, each charge state and isotopic position is considered. But this method did not implement on the peak detection at the peptide or fragment ion level. Sun et al. [17] extended the method of Zhang et al. by developing a model for the whole spectrum considering isotopic pattern and charge state distributions. However, both methods only select the signal peaks based on the intensity information of the observed spectrum. Mcllwain et al. [18] also used Bayesian model to identify isotopic distribution with a dynamic programming algorithm. This model is built to predict the probabilities of each potential isotopic distribution based on length, shape, interdistribution distances, and intradistribution distances. And a dynamic programming algorithm was explored to improve the sensitivity of the classifier and find an optimal sequence of isotopic distribution. But overlapping cases are not taken into account in this method. That would be too restrictive to analyze complex mass spectra. 

In this paper, in order to partially solve the problems of those algorithms above, we present a new algorithm to detect the isotopic clusters of fragment ions and their monoisotopic masses in bottom-up spectra. Considering the complex overlapping cases, isotopic-cluster graphs are constructed to describe the relationship between possible isotopic clusters in range. Nonintensity properties [22] of fragment ions are explored to assist in the determination of monoisotopic peaks in case that those real fragment ions with very low intensity are removed. They are combined with the intensity property of fragment ions in a score function. According to the relationship between isotopic clusters provided by isotopic cluster graphs, each candidate isotopic cluster will be given a score based on the score function. Dynamic programming is adopted to find the highest score path as the optimal arrangement of isotopic clusters with the highest reliability. To test our method, experiments are conducted and compared with YADA [19] which is free available deisotoping software for high-resolution mass spectra.

## 2. Methods

Our deisotoping method is composed of four parts: searching all possible isotopic clusters, constructing isotopic cluster graphs, scoring all possible isotopic clusters and searching paths. The first part aims to find all possible isotopic clusters. The second part is used to describe the relationship between possible isotopic clusters. The third part is used to assess each possible isotopic cluster based on the assumed relationship. The goal of the fourth part is to determine the most possible arrangement of isotopic clusters. 

### 2.1. Searching Possible Isotopic Clusters

**Figure 1:**
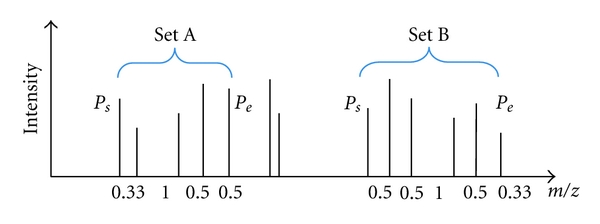
Sets of possible isotopic peaks.

Search starts from the peak with the lowest *m*/*z* value in a spectrum. Firstly, all possible sets of isotopic peaks are generated based on three criterions as follows: each possible set (shown in Figure 1) is composed of several peaks; the number of peaks in each set is no less than 2; the space between any pair of adjacent isotopic peaks in each set is 1.003/*z* (*z* = 1, 2, 3) with an error tolerance 0.01; the starting peak *P*_*s*_ of each set is the first peak which is followed by one peak with the interval 1.003/z (*z* = 1, 2, 3) between them; the ending peak *P*_*e*_ of each set is the last one which follows one peak with the interval 1.003/*z* (*z* = 1, 2, 3) between them. For example, in Figure 1, set *A* consists of five peaks from peak *P*_*s*_ to peak *P*_*e*_. The space between four adjacent peaks is 0.33 (≈1.003/*z*, *z* = 3), 1 (≈1.003/*z*, *z* = 1), 0.5 (≈1.003/*z*, *z* = 2), and 0.5 (≈1.003/*z*, *z* = 2).

**Figure 2:**
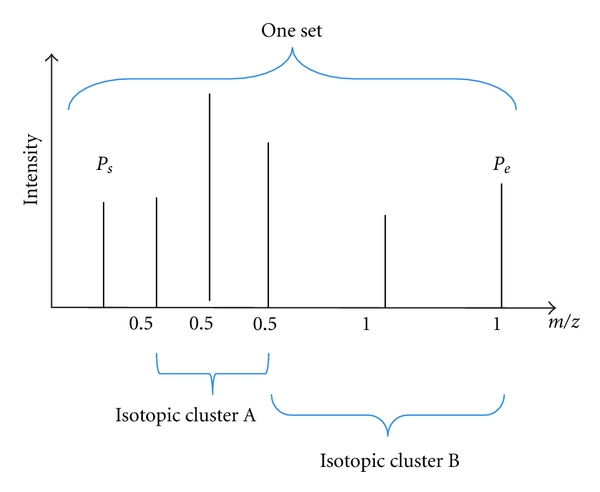
Possible isotopic clusters in one set.

Secondly, in each possible set of isotopic peaks, all candidate isotopic clusters (shown in Figure 2) are searched. Each candidate isotopic cluster searching is followed by two criteria: the range of the number of isotopic peaks for one possible isotopic cluster is from 2 to 3; for one isotopic cluster, the spaces 1.003/*z* (*z* = 1, 2, 3) between each pair of adjacent isotopic peaks are approximately the same. The error tolerance is set to 0.01. In Figure 2, the set includes six peaks. Isotopic cluster *A* and isotopic cluster *B* are two of possible isotopic clusters in the same set. The space between any pair of adjacent peaks in isotopic cluster *A* is 0.5 (≈1.003/*z*, *z* = 2). Isotopic cluster *B* is composed of three peaks of which any pair of adjacent peaks has the same interval 1 (≈1.003/*z*, *z* = 1). 

**Figure 3:**
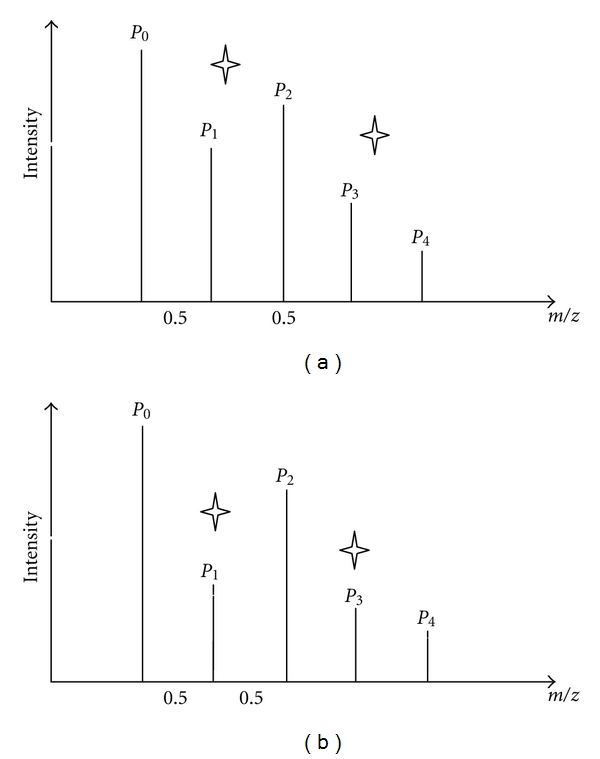
Cases without sharing peaks.

**Figure 4:**
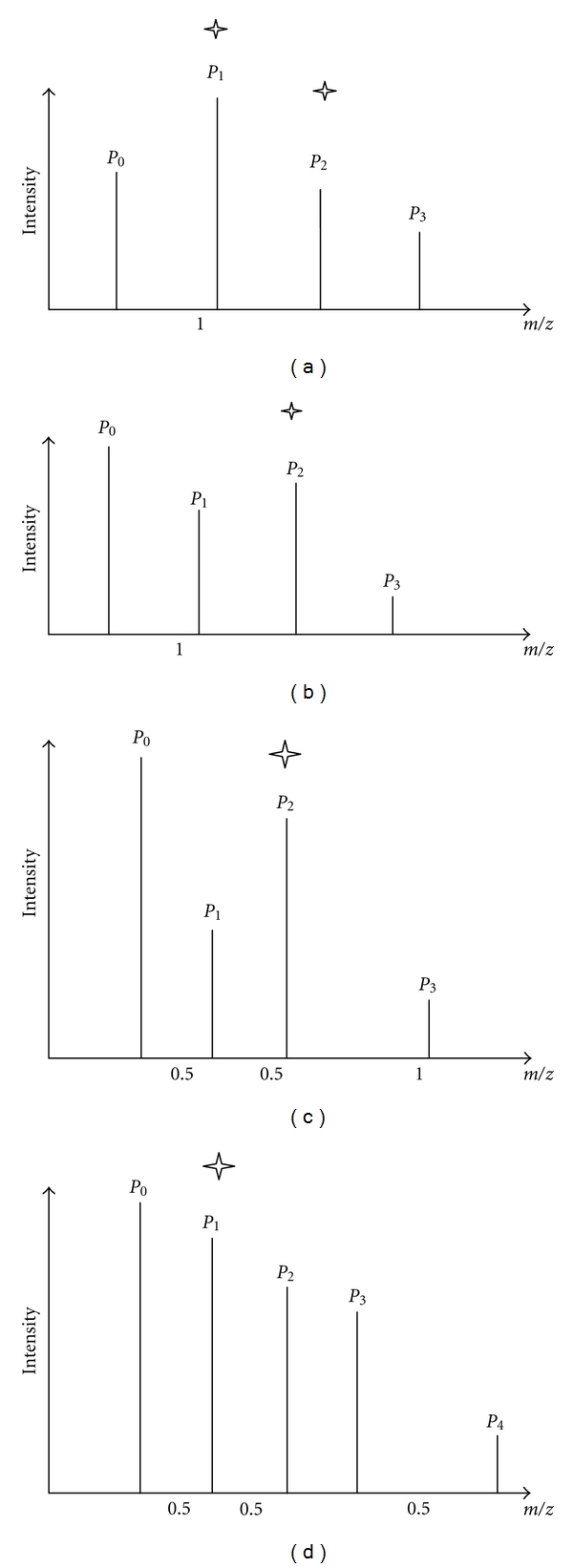
Overlapping cases with sharing peaks.

While searching possible isotopic clusters, several predominant overlapping cases are taken into account. One situation is overlapping cases without sharing peaks (shown in Figure 3). Sets *A* and *B*, of which each includes five peaks *P*_0_ ~ *P*_4_, are two of the examples. In Figure 3(a), one fragment ion is represented by an isotopic cluster composed of *P*_1  _  and *P*_3_. The other isotopic cluster composed of *P*_0_, *P*_2_, and *P*_4_ represents the other fragment ion. There are no sharing peaks in these two isotopic clusters. In Figure 3(b), both *P*_1_, and *P*_3_ are the noise peaks. An isotopic cluster composed of *P*_0_, *P*_2_, and *P*_4_ represents one fragment ion. The other situation is overlapping cases with sharing peaks (shown in Figure 4). In Figure 4(a), one fragment ion with single charge is represented by an isotopic cluster composed of *P*_0_, *P*_1_, and *P*_2_. The other fragment ion with single charge is represented by a different isotopic cluster composed of *P*_1_, *P*_2_, and *P*_3_. Overlapping occurs at *P*_1_ and *P*_2_. In Figure 4(b), two fragment ions with single charge. One is composed of peaks *P*_0_, *P*_1_, and *P*_2_ while the other is composed of peaks *P*_2_, *P*_3_. Overlapping takes place in peak *P*_2_. In Figure 4(c), one fragment ion, represented by the isotopic cluster composed of *P*_0_, *P*_1_, and *P*_2_, is doubly charged. The other fragment ion, represented by the isotopic cluster composed of *P*_2_ and *P*_3_, is singly charged. *P*_2_ is the overlapping peak. In Figure 4(d), one fragment ion, represented by the isotopic cluster composed of *P*_0_, *P*_1_, and *P*_2_, is doubly charged. The other fragment ion, represented by the isotopic cluster composed of *P*_1_, *P*_3_, and *P*_4_, is singly charged. *P*_1_ is the overlapping peak. 

### 2.2. Constructing Isotopic-Cluster Graphs

An isotopic-cluster graph is constructed to describe the predicted relationship between all possible isotopic clusters in each set. Here, the relationship refers to whether or not two connected isotopic clusters overlap and how they overlap. 

**Figure 5:**
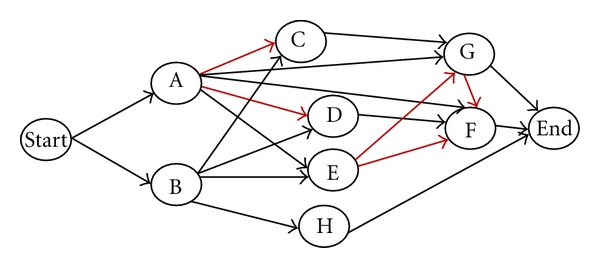
An isotopic-cluster graph.

The source vertex in an isotopic-cluster graph is defined as the starting position, while the sink vertex in an isotopic-cluster graph is defined as the ending position. A vertex in an isotopic-cluster graph is defined as one possible isotopic cluster generated by one possible fragment ion. Two types of edges are constructed in an isotopic-cluster graph: red arcs represent two adjacent isotopic clusters overlap; black arcs represent two adjacent isotopic clusters connecting without overlapping; Figure 5 illustrates how edges in an isotopic-cluster graph are expected to connect the possible isotopic clusters. A black arc is expected to connect one isotopic cluster to the forward isotopic cluster of which the first peak is behind the last peak of the backward isotopic cluster. A red arc is expected to connect two isotopic clusters according to the following rules. (a) The *m*/*z* value of the first peak of the head of an arc is smaller than that of the tail of this arc. (b) If the number of isotopic peaks of the head of an arc is 2, then the second isotopic peak of this head overlaps with the first isotopic peak of the tail of this arc. (c) If the number of isotopic peaks of the head of an arc is 3 and has one sharing peak with the tail of this arc, then the second or third isotopic peak of the head overlaps with the first isotopic peak of the tail. (d) If the number of isotopic peaks of the head, of an arc is 3 and has two sharing peaks with the tail of this arc, then the second and third isotopic peaks of the head respectively overlaps with the first and second isotopic peaks of the tail. The weights of arcs are assigned after assessing possible isotopic clusters by the score function.

### 2.3. Scoring Possible Isotopic Clusters

To avoid peaks of fragment ions with low intensity being removed as noisy peaks by accident, four nonintensity features and one intensity feature of fragment ions are used to assess each possible isotopic cluster. Considering the relationship between adjacent isotopic clusters provided by isotopic-cluster graph, a score function, which is a linear combination of five features, will be explored to score each possible isotopic cluster. To describe these five features, eight variables are defined:

(1) $$\begin{array}{cc} \text{diff}1\left(x,y\right) & =x-y,\\ \text{diff}2\left(x,y\right) & =x-\frac{y+M_{\text{H}}}{2},\\ \text{diff}3\left(x,y\right) & =x-\frac{y+{2\;}^{*}M_{\text{H}}}{3},\\ \text{diff}4\left(x,y\right) & =x-\frac{{2\;}^{*}y+M_{\text{H}}}{3},\\ \text{sum}1\left(x,y\right) & =x+y,\\ \text{sum}2\left(x,y\right) & =x+\frac{y+M_{\text{H}}}{2},\\ \text{sum}3\left(x,y\right) & =x+\frac{y+{2\;}^{*}M_{\text{H}}}{3},\\ \text{sum}4\left(x,y\right) & =x+\frac{{2\;}^{*}y+M_{\text{H}}}{3}, \end{array}$$

where *x* and *y* represent the peaks with *m*/*z* value as *x* and *y*, respectively, in four features (*F*_1_–*F*_4_), *x* is one of the peaks in this isotopic cluster and *y* can be any peaks in a spectrum, and *M*_H_ is the mass of a hydrogen atom. diff1 and sum1 considers that two fragment ions represented by *x* and *y* have the same charge state (*z* = 1, 2, 3); diff2 and sum2 considers that the fragment ion represented by *x* is doubly charged and that represented by *y* is singly charged; diff3 and sum3 considers that the fragment ion represented by *x* is triply charged and that represented by *y* is singly charged; diff4 and sum4 considers that fragment ion represented by *x* is triply charged and that represented by *y* is doubly charged.

To prevent real fragment ions with very low intensity from being removed as noisy peaks, four nonintensity properties of fragment ions which rely on the fragmentation technique, CID, are used to assess the possible isotopic clusters.

The first nonintensity feature (*F*_1_) is based on the number collection of peaks *y* whose mass differences with *x* approximate the residue mass of one of the twenty amino acids. For example, if *x* is one of peaks in an isotopic cluster with *m*/*z* value 100, then the peaks with the *m*/*z* value 171.0788 or 256.1875 in a spectrum are collected as *y* since the relationship between their *m*/*z* values follows one of the formulas below. The differences (171.0788 − 100 = 71.0788, 256.1875 − 100 = 156.1875) are equal to the residue mass of alanine and arginine, respectively:

(2) $$\begin{array}{cc} F_{1} & =|\{y\;|\;\text{abs}\left(\text{diff}1\left(x,y\right)\right)=M_{\text{aa}}+\theta \;\text{or}\;\\ & \;\;\;\;\text{abs}\left(\text{diff}1\left(x,y\right)\right)=\frac{M_{\text{aa}}}{2}+\theta \;\text{or}\\ & \;\;\;\;\text{abs}\left(\text{diff}1\left(x,y\right)\right)=\frac{M_{\text{aa}}}{3}+\theta \;\text{or}\\ & \;\;\;\;\text{abs}\left(\text{diff}2\left(x,y\right)\right)=\frac{M_{\text{aa}}}{2}+\theta \;\text{or}\\ & \;\;\;\;\text{abs}\left(\text{diff}2\left(y,x\right)\right)=\frac{M_{\text{aa}}}{2}+\theta \;\text{or}\\ & \;\;\;\;\text{abs}\left(\text{diff}3\left(x,y\right)\right)=\frac{M_{\text{aa}}}{3}+\theta \;\text{or}\\ & \;\;\;\;\text{abs}\left(\text{diff}3\left(y,x\right)\right)=\frac{M_{\text{aa}}}{3}+\theta \;\text{or}\\ & \;\;\;\;\text{abs}\left(\text{diff}4\left(x,y\right)\right)=\frac{M_{\text{aa}}}{3}+\theta \;\text{or}\\ & \;\;\;\;\text{abs}\left(\text{diff}4\left(y,x\right)\right)=\frac{M_{\text{aa}}}{3}+\theta \}|, \end{array}$$

where abs is the absolute value function, *M*_aa_ is the residue mass of one of twenty amino acids; |·| is the cardinality of a set, the error tolerance *θ* is 0.3 [23]. 

The second nonintensity feature (*F*_2_) is based on the number collection of peaks *y* representing fragment ions that complement with fragment ion represented by *x*.

(3) $$\begin{array}{cc} F_{2} & =|\{y\;|\;\text{sum}1\left(x,y\right)=M+2i+2\times M_{\text{H}}+\theta \;\text{or}\;\;\\ & \;\;\;\;\text{sum}1\left(x,y\right)=\frac{M+2i}{2}+2\times M_{\text{H}}+\theta \;\text{or}\\ & \;\;\;\;\text{sum}1\left(x,y\right)=\frac{M+2i}{3}+2\times M_{\text{H}}+\theta \;\text{or}\\ & \;\;\;\;\text{sum}2\left(x,y\right)=\frac{M+2i}{2}+2\times M_{\text{H}}+\theta \;\text{or}\\ & \;\;\;\;\text{sum}2\left(y,x\right)=\frac{M+2i}{2}+2\times M_{\text{H}}+\theta \;\text{or}\\ & \;\;\;\;\text{sum}3\left(x,y\right)=\frac{M+2i}{3}+2\times M_{\text{H}}+\theta \;\text{or}\\ & \;\;\;\;\text{sum}3\left(y,x\right)=\frac{M+2i}{3}+2\times M_{\text{H}}+\theta \;\text{or}\\ & \;\;\;\;\text{sum}4\left(x,y\right)=\frac{M+2i}{3}+2\times M_{\text{H}}+\theta \;\text{or}\\ & \;\;\;\;\text{sum}4\left(y,x\right)=\;\frac{M+2i}{3}+2\times M_{\text{H}}+\theta \}|, \end{array}$$

where *i* (0,1,…, 3) is the position of peak *x* in its isotopic cluster, *M* is the mass of the neutral precursor ion, and *M*_H_ is the mass of a hydrogen atom. The error tolerance *θ* is 0.3. 

The third nonintensity feature (*F*_3_) considers that the side chains of some amino acids residues of fragment ions can lose a water molecule (H_2_O) or an ammonia molecule (NH_3_). The number of peaks *y* whose mass differences with *x* approximate the mass of a water molecule (H_2_O) or an ammonia molecule (NH_3_) is collected:

(4) $$\begin{array}{cc} F_{3} & =|\{y\;|\;\text{abs}\left(\text{diff}1\left(x,y\right)\right)=M_{{\text{H}}_{2}\text{O}}\;\text{or}\;M_{{\text{NH}}_{3}}+\theta \;\text{or}\;\\ & \;\;\;\;\text{abs}\left(\text{diff}1\left(x,y\right)\right)=\frac{M_{{\text{H}}_{2}\text{O}}}{2}\;\text{or}\;\frac{\;M_{{\text{NH}}_{3}}}{2}+\theta \;\text{or}\\ & \;\;\;\;\text{abs}\left(\text{diff}1\left(x,y\right)\right)=\frac{M_{{\text{H}}_{2}\text{O}}}{3}\;\text{or}\;\frac{M_{{\text{NH}}_{3}}}{3}+\theta \;\text{or}\\ & \;\;\;\;\text{abs}\left(\text{diff}2\left(x,y\right)\right)=\frac{M_{{\text{H}}_{2}\text{O}}}{2}\text{or}\;\frac{M_{{\text{NH}}_{3}}}{2}+\theta \;\text{or}\\ & \;\;\;\;\text{abs}\left(\text{diff}2\left(y,x\right)\right)=\frac{M_{{\text{H}}_{2}\text{O}}}{2}\;\text{or}\;\frac{\;M_{{\text{NH}}_{3}}}{2}+\theta \;\text{or}\\ & \;\;\;\;\text{abs}\left(\text{diff}3\left(x,y\right)\right)=\frac{M_{{\text{H}}_{2}\text{O}}}{3}\;\text{or}\;\frac{M_{{\text{NH}}_{3}}}{3}+\theta \;\text{or}\\ & \;\;\;\;\text{abs}\left(\text{diff}3\left(y,x\right)\right)=\frac{M_{\text{H}2\text{O}}}{3}\;\text{or}\;\frac{\;M_{{\text{NH}}_{3}}}{3}+\theta \;\text{or}\\ & \;\;\;\;\text{abs}\left(\text{diff}4\left(x,y\right)\right)=\frac{M_{{\text{H}}_{2}\text{O}}}{3}\;\text{or}\;\frac{\;M_{{\text{NH}}_{3}}}{3}+\theta \;\text{or}\\ & \;\;\;\;\text{abs}\left(\text{diff}4\left(y,x\right)\right)=\frac{M_{{\text{H}}_{2}\text{O}}}{3}\;\text{or}\frac{\;M_{{\text{NH}}_{3}}}{3}+\theta \}|, \end{array}$$

where *M*_H_2_O_ denotes the mass of a water molecular and *M*_NH_3__ gives the mass of an ammonia molecule. 

The fourth nonintensity feature (*F*_4_) considers two supportive ions *a*-ions and *z*-ions which can be used to indicate the existence of the corresponding *b*-ions and *y*-ions. The number of peaks representing these kinds of supportive ions is collected:

(5) $$\begin{array}{cc} F_{4} & =|\{y\;|\;\text{abs}\left(\text{diff}1\left(x,y\right)\right)=M_{\text{CO}}\;\text{or}\;M_{\text{NH}}+\theta \;\text{or}\;\\ & \;\;\;\;\text{abs}\left(\text{diff}1\left(x,y\right)\right)=\frac{M_{\text{CO}}}{2}\;\text{or}\;\frac{M_{\text{NH}}}{2}+\theta \;\text{or}\\ & \;\;\;\;\text{abs}\left(\text{diff}1\left(x,y\right)\right)=\frac{M_{\text{CO}}}{3}\;\text{or}\;\frac{M_{\text{NH}}}{3}+\theta \;\text{or}\\ & \;\;\;\;\text{abs}\left(\text{diff}2\left(x,y\right)\right)=\frac{M_{\text{CO}}}{2}\;\text{or}\;\frac{M_{\text{NH}}}{2}+\theta \;\text{or}\\ & \;\;\;\;\text{abs}\left(\text{diff}2\left(y,x\right)\right)=\frac{M_{\text{CO}}}{2}\;\text{or}\;\frac{\;M_{\text{NH}}}{2}+\theta \;\text{or}\\ & \;\;\;\;\text{abs}\left(\text{diff}3\left(x,y\right)\right)=\frac{M_{\text{CO}}}{3}\;\text{or}\;\frac{\;M_{\text{NH}}}{3}+\theta \;\text{or}\\ & \;\;\;\;\text{abs}\left(\text{diff}3\left(y,x\right)\right)=\frac{M_{\text{CO}}}{3}\;\text{or}\;\frac{\;M_{\text{NH}}}{3}+\theta \;\text{or}\\ & \;\;\;\;\text{abs}\left(\text{diff}4\left(x,y\right)\right)=\frac{M_{\text{CO}}}{3}\;\text{or}\;\frac{\;M_{\text{NH}}}{3}+\theta \;\text{or}\\ & \;\;\;\;\text{abs}\left(\text{diff}4\left(y,x\right)\right)=\frac{M_{\text{CO}}}{3}\;\text{or}\;\frac{\;M_{\text{NH}}}{3}+\theta \}|, \end{array}$$

where the mass of –CO is denoted by *M*_CO_ and the mass of –NH is denoted by *M*_NH_. 

The intensity feature (*F*_5_) determines if the experimental isotopic distribution of one possible isotopic cluster matches with the theoretical isotopic distribution or not with the consideration of the relationship between adjacent isotopic clusters in the graph.

Based on the natural abundance of the composition elements in one ion, the theoretical isotopic distribution of this ion can be predicted. However, the fragment ion represented by one isotopic cluster is unknown in a tandem mass spectrum. Thus, the theoretical isotopic distribution cannot be predicted precisely. Three extreme cases of the composition of peptide fragment ions are used to estimate the maximum, the mean, and the minimal of the theoretical isotopic pattern: one is composed of all phenylalanine C_9_H_9_NO [24]; one is composed of an updated version of Averigine C_4.949_H_7.833_O_1.473_N_1.361_S_0.038_ [25]; one consists of all aspartic C_4_H_5_NO_3_ [24]. Assume that a particular molecular mass is known, and then the number of phenylalanine units, Averigine units, and aspartic units of this molecule can be calculated. Then, the element composition of this molecule can be acquired. Besides, the relative natural abundance of each element C, H, N, and O is already known. Based on the information above, the maximum, mean, and minimum theoretical isotopic distribution of an ion with a particular mass can be predicted:

(6) $$F_{5}=\left|\left\{\begin{array}{c} y^{\prime }\;|\;\frac{\min\left(\left|E_{i}-{\left(T_{\min}\right)}_{i}\right|,\left|E_{i}-{\left(T_{\max}\right)}_{i}\right|\right)}{{\left(T_{\text{mean}}\right)}_{i}}\leq \text{threshold}\;\text{or}\\ \frac{\min\left(\left|\left(E_{i}-{\left(T_{\text{mean}}^{\prime }\right)}_{i}\right)-{\left(T_{\min}\right)}_{i}\right|,\left|\left(E_{i}-\left({T_{\text{mean}}^{\prime }}_{\;}\right)\right)-{\left(T_{\max}\right)}_{i}\right|\right)}{{\left(T_{\text{mean}}\right)}_{i}}\leq \text{threshold} \end{array}\right\}\right|,$$

where the first formula is for an isotopic cluster that has no sharing peaks with others, the second formula is for an isotopic clusters that has sharing peaks with others; *E*_*i*_ is the experimental intensity of peak *I*, (*T*_min⁡_)_*i*_ is the minimum theoretical intensity of peak *i*, (*T*_max⁡_)_*i*_ is the maximum theoretical intensity of peak *I*, (*T*_mean_)_*i*_ is the mean theoretical intensity of peak *i*, (*T*_mean_′)_*i*_ is the mean theoretical intensity of the other isotopic cluster which is overlapped with this isotopic cluster, *i* (1,…, 3) is the order of peak *x* in this isotopic cluster. Threshold is set as 0.3. Here in *F*_5_, *y*′ and *x*′  belong to the same assumed isotopic cluster. *x*′ is the first peak of the isotopic cluster, and *y*′ is the rest of this isotopic cluster.

To thoroughly assess each possible isotopic cluster, those five features above are combined in a score function as follows:

(7) $$\text{score}=\omega_{1}\times F_{1}+\omega_{2}\times F_{2}+\omega_{3}\times F_{3}+\omega_{4}\times F_{4}+\omega_{5}\times F_{5},$$

where *F*_*i*_ (*i* = 1,…, 5) is the value of each feature *ω*_*i*_ (*i* = 1,…, 5) are the coefficients which are estimated by using linear discriminative analysis (LDA) [26] with the training dataset. We get *ω*_1_ = 0.8; *ω*_2_ = 0.5; *ω*_3_, *ω*_4_, and *ω*_5_ = 0.1.

Each pair of adjacent possible isotopic clusters in one isotopic-cluster graph will be assessed by the score function at the same time. Based on their relationship in the graph, each peak in one possible isotopic cluster will be given a score. The sum score of all peaks in each possible isotopic cluster is considered as the score of this possible isotopic cluster. The same peak in different possible isotopic clusters can get different scores due to the facts that (a) its charge state depends on the interval of adjacent peaks in the isotopic cluster it belongs to; (b) its position order is different in different isotopic clusters; (c) the relationships between its isotopic cluster and adjacent isotopic cluster are different. The scores of correct isotopic clusters are expected to be higher than that of incorrect ones.

**Figure 6:**
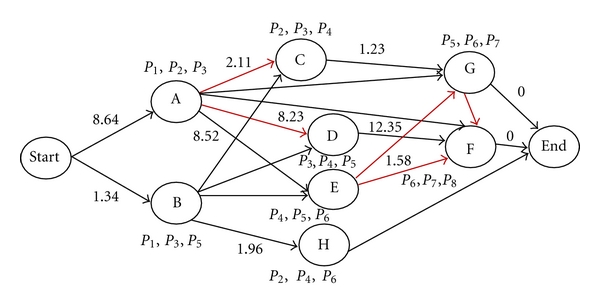
An isotopic-cluster graph with assigned weights.

The weight of each arc of an isotopic-cluster graph (Figure 6) is assigned based on the calculated score of the backward isotopic cluster in each pair of connected isotopic clusters. If an isotopic cluster connects with the ending vertex, then the weight between them is assigned as zero. The larger the weight between two connected isotopic clusters is, the more reliable the assumed relationship between them is.

### 2.4. Search Paths

A path in a directed acyclic graph is defined as a sequence of vertices without repeated vertices. The score of a path in the isotopic-cluster graph is the sum of the weights of all edges of this path. The higher the total score of one path is, the more reliably the isotopic clusters are detected. The paths with the highest score in an isotopic-cluster graph are those that cover edges with high weights. The isotopic clusters of fragment ions are determined by searching for optimal paths in the isotopic-cluster graphs. To identify the isotopic clusters, dynamic programming will be adopted to find the path with the highest score in each isotopic-cluster graph. 

## 3. Experimental Dataset

### 3.1. Training Dataset

To estimate the weights of each feature in the score function, a training dataset is constructed based on dataset in [27]. The sample from *Escherichia Coli* after being digested with trypsin was analyzed by *μ*LC-MS/MS on a ThermoFinnigan Orbitrap LTQ mass spectrometer, yielding a total of 112329 mass spectra [27]. Of them, 1208 high-confidence peptide-spectrum matches MS/MS dataset generated by some algorithms [28–31] was used to generate the training dataset. The thresholds for getting those high-confidence peptide-spectrum matches were set with an FDR of 1%. The charge range of spectra is from 1 to 2 while the mass range of spectra is from 0 to 2000 Da. The training dataset consists of two groups: one group with incorrect isotopic clusters and the other group with correct isotopic clusters. Since the theoretical peptide sequences of those 1208 spectra is known, we used Peptide Fragmentation Modeller [32] to generate the theoretical fragment ions for each spectrum. Meantime, MS-Deconv software [33] processed those 1208 spectra and generated a list of isotopic clusters for each spectrum. Then, the MS-Deconv's outputs are compared with the corresponding theoretical spectra. The matched isotopic clusters are grouped as correct isotopic clusters. The rest of possible isotopic clusters of the original spectra are grouped as incorrect isotopic clusters.

### 3.2. Testing Dataset

To evaluate the performance of our deisotoping method, we used one MS/MS dataset [34] in FT2 format consisting of 3273 bottom-up spectra which is derived from the digestion of *R. palustris CGA010* strain. This dataset was analyzed with a two-dimensional liquid chromatography-tandem mass spectrometry analysis (2D LC-MS/MS). Peptides eluted from the microcapillary columns were electrosprayed into an LTQ-Orbitrap mass spectrometer (ThermoFisher Scientific, San Jose, CA, USA). The RAW format outputs of LTQ-Orbitrap mass spectrometer were converted to FT2 format. The charge range of spectra is from 1 to 3. The mass range of spectra is from 600 to 7000 Da. Our deisotoping method is compatible with the MGF file, and YADA software can deal with the MS2 file. Thus, we wrote two MATLAB scripts to convert the testing dataset from FT2 format to individual MGF file and MS2 file, respectively.

## 4. Results and Discussion

### 4.1. Compared with YADA and MS-Deconv

In this section, we compared my method with two pieces of software YADA and MS-Deconv. Here, YADA software mainly deisotopes high-resolution middle-down spectra, but can process bottom-up mass spectra as well. MS-Deconv can decharge and deisotope complex tandem mass spectra as well. This evaluation was processed from two aspects by applying them to 1208 bottom-up spectra (the training data set): (a) to see if peptides and proteins identification get better from the number of interpreted spectra and the score of interpreted spectra by Mascot [35]; (b) to see if more fragment ions can be detected from the number of real monoisotopic masses of fragment ions.

#### 4.1.1. Identification of Peptides and Proteins

To assess the performance of peptide and protein identification, the online Mascot searching was employed to interpret the dataset processed by our deisotoping method, YADA and MS-Deconv. Before Mascot searching, we wrote two MATLAB scripts to convert the YADA's output from the MS2 file to MGF file and convert the MS-Deconv's output from ENV files to MGF files, respectively. The cysteine residues were set to be carboxamidomethyled as a fixed modification, and methionine residues were set to be oxidized as a variable modification. All the searches were processed in the SWISS-PROT database with one missed trypsin cleavages allowed. The tolerance for the peptide mass is 1.2 Da and for the fragment mass is 0.6 Da. In this study, the peptides are considered to be interpreted by Mascot searching engine with an FDR of 1%.

The more peptides and proteins interpreted by Mascot after being processed, the better the effect of the deisotoping method. Therefore, we used the number of interpreted peptides and proteins to assess the performance. The search results in Table 1 show that 281, 273, and 259 peptides are interpreted while a total of 196, 181, and 172 proteins are identified from the same spectra dataset processed by our method, YADA, and MS-Deconv, respectively.

**Figure 7:**
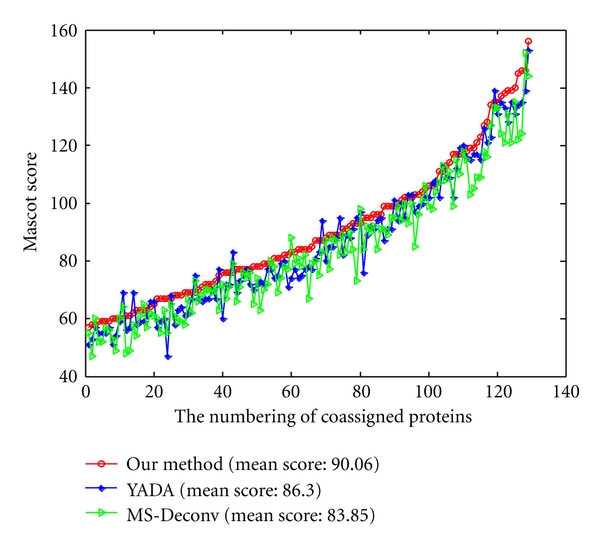
The Mascot scores on 129 proteins which are coassigned by data processed by our method (red line), data processed by YADA (blue line), and data processed by MS-Deconv (green line).

**Figure 8:**
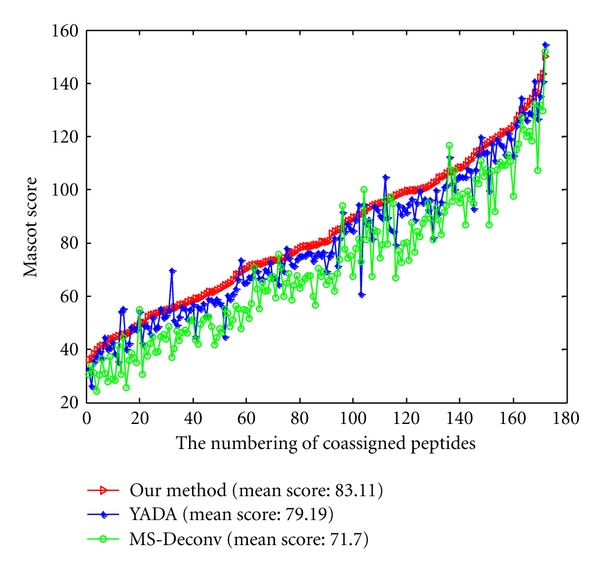
The Mascot scores on 172 peptides which are coassigned by data processed by our method (red line), data processed by YADA (blue line), and data processed by MS-Deconv (green line).

The higher the Mascot score is, the higher reliability the peptide and protein identifications are. To ensure the fairness, the Mascot score comparisons are processed on 129 coassigned proteins (Figure 7) and 172 coassigned peptides (Figure 8) from data processed by three methods with the same parameters. From Figure 7, although the Mascot score of a few proteins from processed data by YADA and MS-Deconv is greater than from our method, the mean Mascot score of interpreted proteins from the processed data by our method are increased by 4.3% and 7.4% than that from processed data by YADA and MS-Deconv, respectively. From Figure 8, although the Mascot score of a few peptides from processed data by YADA and MS-Deconv is greater than that from our method, the mean Mascot scores of the interpreted peptides of the data processed by our method has 4.95% and 15.9% improvement over those processed data by YADA and MS-Deconv, respectively. From the results above, the Mascot searches on the data processed by our method is more reliable than those by YADA and MS-Deconv.

#### 4.1.2. Determination of Monoisotopic Peaks

The more real monoisotopic peaks detected by the deisotoping method, the more important information of fragment ions obtained and the more accuracy of peptide identification. To compare the performance of the real monoisotopic masses determination on the processed data by our method, YADA, and MS-Deconv, the *F*-score analysis is introduced.

Based on each known theoretical peptide sequence of 1208 spectra, Peptide Fragmentation Modeller generated a list of theoretical fragment ions, including *a*, *b*, *c*, *x*, *y*, *z* and neutral ions. After that, a spectrum processed by our method, YADA, and MS-Deconv was compared with its corresponding theoretical spectrum. If the difference between a peak in each experimental spectrum and a peak in its corresponding theoretical spectrum is within a given error tolerance, the peak in the experimental spectrum is regarded as a true positive (TP), and otherwise it is regarded as a false positive (FP). If the differences between a peak in theoretical spectrum and any peak in its corresponding experimental spectrum are beyond a given error tolerance, the peak in theoretical spectrum is regarded as a false negative (FN). We used the *F*-score to investigate the performance of our method, YADA, and MS-Deconv. The *F*-score is computed by considering both the precision and the recall:

(8) $$F=2*\frac{\text{precision}*\text{recall}}{\text{precision}+\text{recall}},$$

where precision is defined as TP/(TP + FP) and recall, also called sensitivity, is defined as TP/(TP+FN).

**Figure 9:**
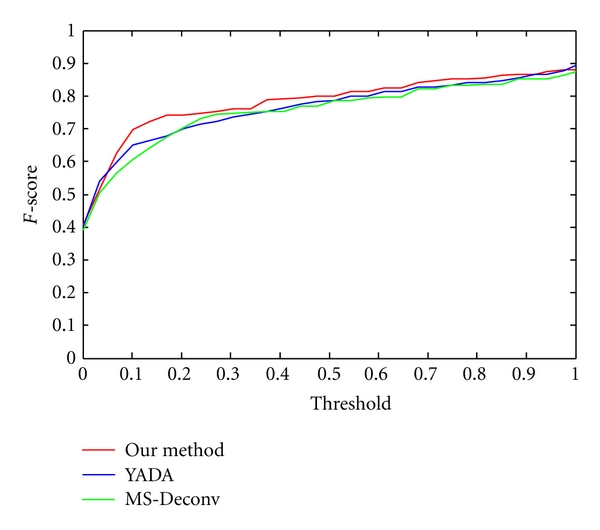
The *F*-scores of 172 coassigned spectra from our method's outputs (red line), YADA's outputs (blue line), and MS-Deconv's output (green line).

A series of mass error tolerances ranging from 0 to 1 Da were selected while comparing an experimental spectrum with a theoretical spectrum. With different mass error tolerances, we got *F*-score curves shown in Figure 9 for three methods.

For fairness, the calculated *F*-scores were compared on 172 coassigned spectra of our method's outputs, YADA's outputs, and MS-Deconv's output. It can be observed from Figure 9 that under different mass error tolerances almost all *F*-scores from our outputs are greater than those from YADA's outputs and MS-Deconv's output. It suggests that our method is more accurate than YADA and MS-Deconv in the detection of real monoisotopic peaks.

### 4.2. Performance on the Testing Data Set

In this section, to investigate the performance of our method further, it was compared with the software YADA on testing data set from the same aspects as the last section.

#### 4.2.1. Identification of Peptides and Proteins

To investigate the performance of peptide and protein identification, the online Mascot searching was employed to interpret the raw MS/MS dataset, the dataset processed by YADA, and that by our deisotoping method. The searching parameters are set as same as the last section.

**Figure 10:**
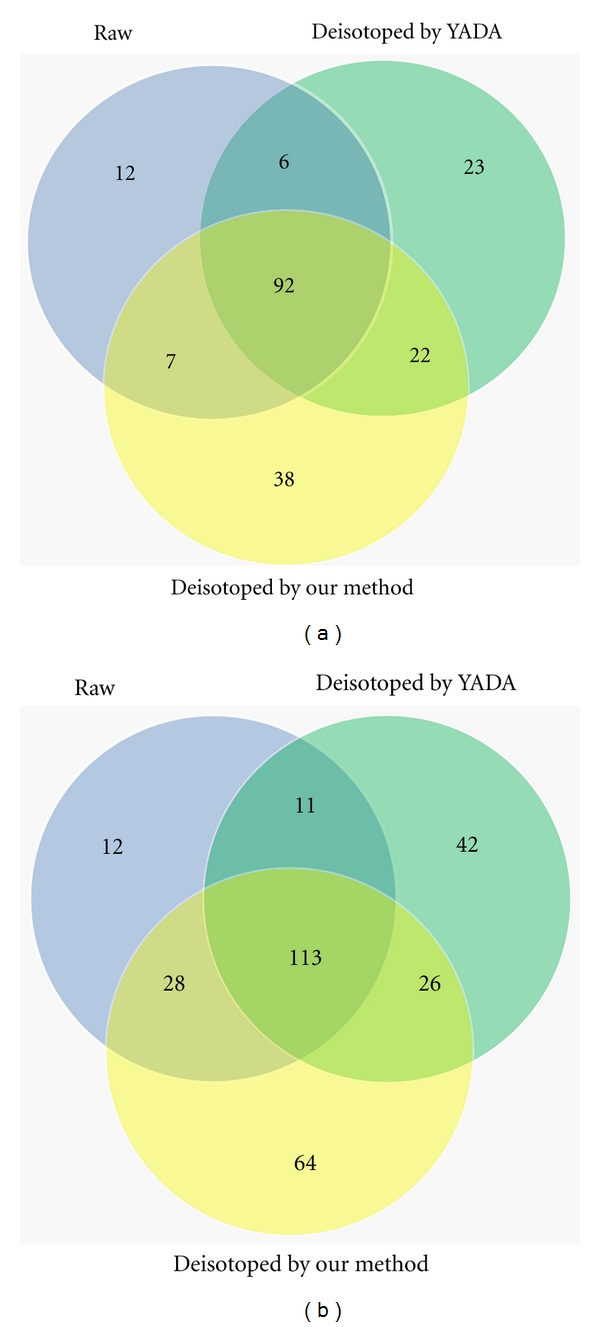
Comparison of identified proteins (a) and peptides (b) from the raw data, deisotoped data by our method and by YADA.

The effect of the deisotoping method can be indicated from the number of peptides and proteins interpreted by Mascot.  Table 2 shows the number of the interpreted peptides and proteins in raw data, the processed data by YADA, and that by our method. From this table, we can see that the number of interpreted proteins increased by 22.22%  ( = (143 − 117)/117) for the data processed by YADA and 35.90% ( = (159 − 117)/117) by our method. It also shows that our method can improve the number of identified peptides by 20.31% ( = (231 − 192)/192) compared to YADA and 40.85% ( = (231 − 164)/164) compared to the raw data. Both the increasing rates of the identified proteins and peptides after using our method are greater than those after applying YADA. In addition, from Figure 10(a), up to 79.72% ( = (92 + 22)/(92 + 22 + 23 + 6)) interpreted proteins from the processed data by YADA, and 84.62% ( = (92 + 7)/(92 + 7 + 12 + 6)) for the raw data are also identified from the processed data by our method. Moreover, 23.90% ( = 38/159) newly identified proteins only comes from the data processed by our method.  Figure 10(b) shows that up to 72.40% ( = (113 + 26)/(11 + 113 + 42 + 26)) interpreted peptides from the processed data by YADA, and 85.98% ( = (28 + 113)/(28 + 113 + 12 + 11)) for the raw data are also identified from the processed data by our method. 27.71% ( = 64/231) are only identified by our method. From the results above, more peptides and proteins are identified by Mascot from the data processed by our method than that from the raw data and the data processed by YADA. It indicates that our method has better effect on the Mascot search than YADA.

**Figure 11:**
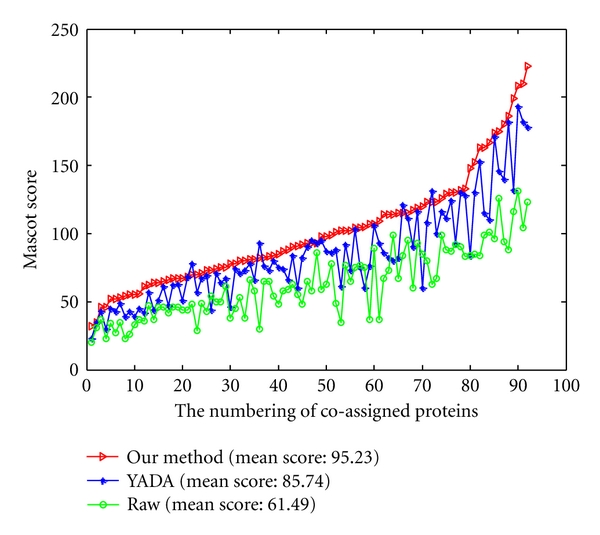
The Mascot scores on the proteins which are coassigned by raw data (green line), data processed by YADA (blue line) and by our method (red line).

The reliability of the peptide and protein identifications is assessed based on the Mascot score. To ensure the fairness, the Mascot scores comparison is processed on the coassigned proteins and peptides from the raw data and two processed data with the same parameters.  Figure 11 shows the Mascot scores of the 92 overlapped proteins from raw data and two processed data. Compared with raw data, the mean Mascot score of the interpreted proteins from YADA processed data and from our method processed data is increased by 41.06% ( = (86.74 − 61.49)/61.49) and 54.87% ( = (95.23 − 61.49)/61.49), respectively. The result indicates that the reliability of protein identification increases by applying both YADA and our method. However, our method performs better than YADA with the increasing rate of 9.79% ( = (95.23 − 86.74)/86.74).

**Figure 12:**
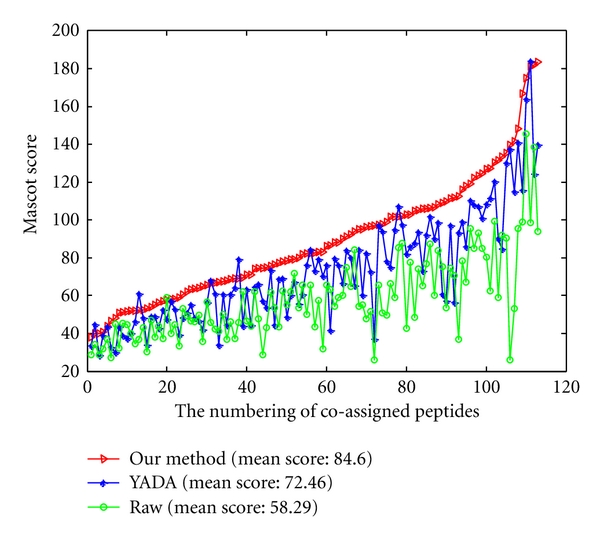
The Mascot scores on the peptides which are coassigned by raw data (green line), data processed by YADA (blue line) and by our method (red line).

The Mascot scores of 113 cointerpreted peptides from raw data and two processed data were compared in Figure 12. As we can see in this figure, both the curves from YADA and our method are higher than the curve representing the mascot score of raw data. The mean Mascot scores of the interpreted peptides from YADA processed data and from the data processed by our method are increased by 24.31% ( = (72.46 − 58.29)/58.29) and 45.14% ( = (84.60 − 58.29)/58.29) over those of the raw data. Furthermore, our method has 16.75% ( = (84.60 − 72.46)/72.46) improvement over YADA. From the results above, the Mascot searches on the data processed by our method is more reliable than that on the raw data and data processed by YADA.

Moreover, in order to assess the effect of deisotoping on the speed of the Mascot analysis, the Mascot searching time (in seconds) is roughly recorded. For the raw data, the searching time is around 121 s. For the data processed by YADA software, the Mascot searching time is reduced to 75 s. After being processed by our deisotoping method, the searching time is decreased to 69 s. The results illustrate that our method can reduce the Mascot searching time by providing Mascot search engine with shorter lists of more real monoisotopic masses compared to raw data.

#### 4.2.2. Determination of Monoisotopic Peaks

To evaluate the performance for determining real monoisotopic masses and compare our method with YADA, we used the *F*-score analysis as in Section 4.1.

**Figure 13:**
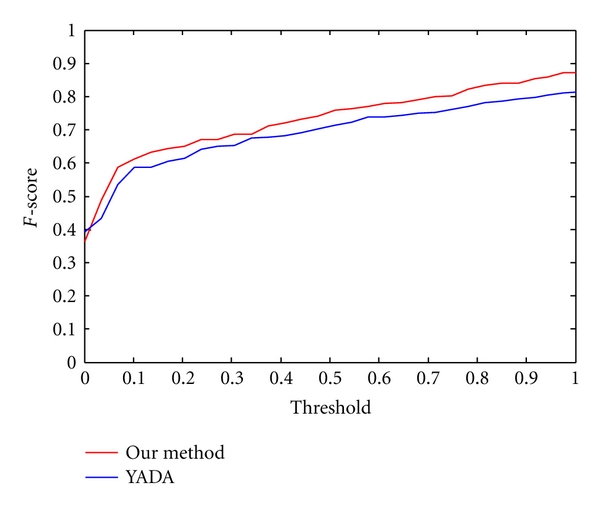
The *F*-scores of 139 coassigned spectra from our method's outputs (red line) and YADA's outputs (blue line).

We firstly generated the theoretical peptide sequences for the testing dataset (3273 spectra) by PEAKS [36]. Of PEAKS' output, 2363 theoretical peptide sequences whose average local confidences are larger than 60% were selected. Then, based on each theoretical peptide sequence, Peptide Fragmentation Modeller generated a list of theoretical fragment ions, including *a*, *b*, *c*, *x*, *y*, *z* and neutral ions. After that, each spectrum of our output and YADA's output was compared with each corresponding theoretical spectrum. A series of mass error tolerances ranging from 0 to 1 Da were selected while comparing experimental spectrum with theoretical spectrum. We used the *F*-score (formula 7) to investigate the performance of our deisotoping method and YADA. For fairness, the calculated *F*-scores (shown in Figure 13) were compared on 139 coassigned spectra by YADA's outputs and our method's outputs. It can be observed from Figure 13 that under different mass error tolerances almost all *F*-scores from our outputs are greater than those from YADA's outputs. It suggests that our method is more accurate than YADA in the detection of real monoisotopic peaks.

## 5. Conclusion

This paper has presented a deisotoping algorithm for bottom-up spectra to increase the accuracy of monoisotopic mass determination of fragment ions. The algorithm takes overlapping cases into account by firstly constructing isotopic-cluster graphs which describe the relationship between possible isotopic clusters. Based on the assumed relationships in the graphs, all possible isotopic clusters are evaluated by a score function which combines nonintensity and intensity features of fragment ions. This method could help retain fragment ions with very low intensity in spectra. The experimental results on two data sets have indeed indicated that our method performs better in deisotoping compared with YADA and MS-Deconv software from three aspects: (1) the number of interpreted proteins and peptides from the dataset processed by our deisotoping method is larger than that from raw data, data processed by YADA and MS-Deconv, (2) the peptide and protein identifications from the data processed by our method are more reliable than those from the other two kinds of software, and (3) the *F*-scores of our method are greater than those of other two kinds of software. In the future, we will test our method on more mass spectral datasets.

## Figures and Tables

**Table 1:** Numbers of peptides and proteins identified by Mascot from data (1208 spectra) processed by our method, YADA, and MS-Deconv.

	Data processed by MS-Deconv	Data processed by YADA	Data processed by our method
Proteins	172	181	196
Peptides	259	273	281

**Table 2:** Numbers of peptides and proteins identified by Mascot from the raw data (3273 spectra) and processed data by our method and YADA.

	Raw data	Data processed by YADA	Data processed by our method
Proteins	117	143	159
Peptides	164	192	231

## References

[1] Dass C (2007). *Fundamentals of Contemporary Mass Spectrometry*.

[2] Sun Y, Zhang J, Braga-Neto U, Dougherty ER (2010). BPDA—a Bayesian peptide detection algorithm for mass spectrometry. *BMC Bioinformatics*.

[3] Zhang J, Xu D, Gao W, Lin G, He S (2009). Isotope pattern vector based tandem mass spectral data calibration for improved peptide and protein identification. *Rapid Communications in Mass Spectrometry*.

[4] Zhang J, He S, Ling CX, Cao X, Zeng R, Gao W (2008). PeakSelect: preprocessing tandem mass spectra for better peptide identification. *Rapid Communications in Mass Spectrometry*.

[5] Horn DM, Zubarev RA, McLafferty FW (2000). Automated reduction and interpretation of high resolution electrospray mass spectra of large molecules. *Journal of the American Society for Mass Spectrometry*.

[6] Kaur P, O’Connor PB (2006). Algorithms for automatic interpretation of high resolution mass spectra. *Journal of the American Society for Mass Spectrometry*.

[7] Noy K, Fasulo D (2007). Improved model-based, platform-independent feature extraction for mass spectrometry. *Bioinformatics*.

[8] Jaitly N, Mayampurath A, Littlefield K, Adkins JN, Anderson GA, Smith RD (2009). Decon2LS: an open-source software package for automated processing and visualization of high resolution mass spectrometry data. *BMC Bioinformatics*.

[9] Sturm M, Bertsch A, Gröpl C (2008). OpenMS—an open-source software framework for mass spectrometry. *BMC Bioinformatics*.

[10] Masselon C, Paša-Tolić L, Lee SW (2003). Identification of tryptic peptides from large databases using multiplexed tandem mass spectrometry: simulations and experimental results. *Proteomics*.

[11] Senko MW, Beu SC, McLafferty FW (1995). Automated assignment of charge states from resolved isotopic peaks for multiply charged ions. *Journal of the American Society for Mass Spectrometry*.

[12] Li XJ, Yi EC, Kemp CJ, Zhang H, Aebersold R (2005). A software suite for the generation and comparison of peptide arrays from sets of data collected by liquid chromatography-mass spectrometry. *Molecular and Cellular Proteomics*.

[13] Samuelsson J, Dalevi D, Levander F, Rögnvaldsson T (2004). Modular, scriptable and automated analysis tools for high-throughput peptide mass fingerprinting. *Bioinformatics*.

[14] Du P, Angeletti RH (2006). Automatic deconvolution of isotope-resolved mass spectra using variable selection and quantized peptide mass distribution. *Analytical Chemistry*.

[15] Renard BY, Kirchner M, Steen H, Steen JAJ, Hamprecht FA (2008). NITPICK: peak identification for mass spectrometry data. *BMC Bioinformatics*.

[16] Zhang J, Wang H, Suffredini A Bayesian peak detection for pro-TOF MS MALDI data.

[17] Sun Y, Zhang J, Braga-Neto U, Dougherty ER (2010). BPDA—a Bayesian peptide detection algorithm for mass spectrometry. *BMC Bioinformatics*.

[18] McIlwain S, Page D, Huttlin EL, Sussman MR (2007). Using dynamic programming to create isotopic distribution maps from mass spectra. *Bioinformatics*.

[19] Carvalho PC, Xu T, Han X, Cociorva D, Barbosa VC, Yates JR (2009). YADA: a tool for taking the most out of high-resolution spectra. *Bioinformatics*.

[20] Yergey JA (1983). A general approach to calculating isotopic distributions for mass spectrometry. *International Journal of Mass Spectrometry and Ion Physics*.

[21] Rockwood AL, Van Orden SL, Smith RD (1995). Rapid calculation of isotope distributions. *Analytical Chemistry*.

[22] Wu FX, Gagné P, Droit A, Poirier GG (2008). Quality assessment of peptide tandem mass spectra. *BMC Bioinformatics*.

[23] Wong JWH, Sullivan MJ, Cartwright HM, Cagney G (2007). msmsEval: tandem mass spectral quality assignment for high-throughput proteomics. *BMC Bioinformatics*.

[24] Park K, Joo YY, Lee S (2008). Isotopic peak intensity ratio based algorithm for determination of isotopic clusters and monoisotopic masses of polypeptides from high-resolution mass spectrometric data. *Analytical Chemistry*.

[25] Barbarini N, Magni P (2010). Accurate peak list extraction from proteomic mass spectra for identification and profiling studies. *BMC Bioinformatics*.

[26] Lin W, Wu FX, Shi J, Ding J, Zhang W (2010). An adaptive approach to denoising tandem mass spectra. *Proceedings of the IEEE International Conference on Bioinformatics and Biomedicine Workshops (BIBMW '10)*.

[27] Klammer AA, Reynolds SM, Bilmes JA, Maccoss MJ, Noble WS (2008). Modeling peptide fragmentation with dynamic Bayesian networks for peptide identification. *Bioinformatics*.

[28] Eng JK, McCormack AL, Yates JR (1994). An approach to correlate tandem mass spectral data of peptides with amino acid sequences in a protein database. *Journal of the American Society for Mass Spectrometry*.

[29] Geer LY, Markey SP, Kowalak JA (2004). Open mass spectrometry search algorithm. *Journal of Proteome Research*.

[30] Zhang N, Aebersold R, Schwikowski B (2002). ProbID: a probabilistic algorithm to identify peptides through sequence database searching using tandem mass spectral data. *Proteomics*.

[31] Frank A, Pevzner P (2005). PepNovo: de novo peptide sequencing via probabilistic network modeling. *Analytical Chemistry*.

[32] Peptide fragmentation modeller. http://omics.pnl.gov/software/PeptideFragmentationModeller.php.

[33] Liu X, Inbar Y, Dorrestein PC (2010). Deconvolution and database search of complex tandem mass spectra of intact proteins: a combinatorial approach. *Molecular and Cellular Proteomics*.

[34] Pan C, Park BH, McDonald WH (2010). A high-throughput de novo sequencing approach for shotgun proteomics using high-resolution tandem mass spectrometry. *BMC Bioinformatics*.

[35] Perkins DN, Pappin DJC, Creasy DM, Cottrell JS (1999). Probability-based protein identification by searching sequence databases using mass spectrometry data. *Electrophoresis*.

[36] Ma B, Zhang K, Hendrie C (2003). PEAKS: powerful software for peptide de novo sequencing by tandem mass spectrometry. *Rapid Communications in Mass Spectrometry*.

